# Evaluation of synergistic therapeutic effect of shark cartilage extract with artemisinin and glucantime on visceral leishmaniasis in BALB/c mice

**DOI:** 10.22038/ijbms.2018.31124.7504

**Published:** 2019-02

**Authors:** Soheila Molaie, Fatemeh Ghaffarifar, Abdohosein Dalimi, Mohammad Hassan Zuhair, Zohreh Sharifi

**Affiliations:** 1 Department of Parasitology, School of Medical Sciences, Tarbiat Modares University, Tehran, Iran; 2 Department of Immunology, School of Medical Sciences, Tarbiat Modares University, Tehran, Iran; 3 Department of Virology, Iranian Blood Transfusion, Tehran, Iran; 4 Deputy of Research, Ardabil University of Medical Sciences, Ardabil, Iran

**Keywords:** Artemisinin, BALB/c mice, Glucantim, Shark cartilage extract, Synergism, Visceral leishmaniasis

## Abstract

**Objective(s):**

Because leishmaniasis is related to the impaired functioning of T-cells, the use of an immunomodulator can increase the efﬁcacy of antileishmanial therapy in visceral leishmaniasis. In this study, we used shark cartilage extract with artemisinin and glucantime against visceral leishmaniasis in BALB/c mice, and evaluated the synergistic therapeutic effect.

**Materials and Methods:**

The culturing method and quantitative real-time PCR by using the kDNA gene was used to detect parasite loads in the spleen and liver. INF-γ and IL-4 cytokine levels and survival rates were assayed.

**Results:**

The drug therapy with target drugs reduced parasite burden in the spleen and liver significantly. Although parasite burden was lower in the artemisinin treated group than in the glucantime treated group (*P*<0.05). The mice survival rate records, throughout the experimental period, showed highly significant survival rates in the test groups compared to the control group (*P*<0.001). The results of cytokine assay in mice treated with glucantime-shark cartilage extract combination indicated significant increases of IFNγ and IL-4 (*P*<0.05). Although the increase of IFNγ was more notable than IL-4. The synergistic therapeutic effect is shown in all groups except in the group treated with shark cartilage extract-artemisinin combination. The IFN-γ in glucantime-shark cartilage extract combination treated group was higher than in other groups (*P*<0.05). The survival rate in this group was more than in other groups too (*P*<0.05).

**Conclusion:**

Combination therapy with shark cartilage extract as an immunomodulator can increase antileishmanial effects of antimony drugs in VL treatment.

## Introduction

Leishmaniasis is an infectious neglected tropical disease with different clinical features ranging from benign cutaneous scars in leishmaniasis (CL) to chronic ulcerating mucocutaneous leishmaniasis (MCL) or acute visceral form in visceral leishmaniasis (VL). The agent of visceral leishmaniasis is *Leishmania donovani *complex, including *Leishmania donovani*, *Leishmania infantum, *and *Leishmania chagasi *(1)*. *In the Mediterranean region, infection is caused by *L. infantum, *which creates an important health problem, especially in infants (2, 3). In the absence of an effective vaccine, the control of the disease is based on chemotherapy. Pentavalent antimonials (4), paromomycin (5), deoxycholate and sitamaquine (6), amphotericin B (7), and miltefosine (8) are the current drugs in visceral leishmaniasis treatment. These drugs, however, have shown serious side effects and drug resistance, besides being expensive, teratogen, and of long half-time in some countries (9). Given these problems, development of new anti-leishmanial compounds would be necessary. In recent years, plant compounds such as alkaloids, terpenoids, flavonoids, saponins, quinones, and chalcones were used to treat leishmaniasis (10, 11). Artemisinin is a sesquiterpene trioxane lactone, produced in glandular trichomes (GLTs) of *Artemisia annua L.* being currently the best drug against malaria (12), with wide spectrum anti-leishmanial activity against several leishmania species in both *in vitro *(13-17) and *in vivo *experimentations on animal models (18, 19). On the other hand, when T-cell function is interrupted and macrophages are unable to initiate phagocytosis of leishmania parasites, leishmaniasis occurs, so the modulation of patient immune responses seems to be necessary (20). Immune responses in murine models are especially mediated by T lymphocytes and T helper (Th1) and Th2 cells and can be indicated by cytokine discharge. Th1 cells secrete Interferon-gamma (IFN-γ), the essential cytokine, to control visceral infection, associated with the parasite-specific cellular immune responses, while Th2 cells secrete IL-4, which boosts antibody responses (21). Additionally, studies have shown that artemisinin plays a bilateral role in leishmaniasis, a direct antiparasitic effect to increase production of NO and iNOS expression in uninfected macrophages and an indirect immunomodulatory effect with increasing the emancipation of Th1 cytokines (19).

In recent studies, the immunomodulatory activity of shark cartilage extract has been noticed (22). Shark cartilage contains several chemicals such as proteins, glycoproteins, and glycosaminoglycans (23), and has shown several properties in different disorders: angiogenesis inhibitor in the treatment of cancer (24), as a joint lubricant in arthritis (25), treatment of psoriasis and diabetic retinopathy (26), and as an immunomodulator induces Th1 type inflammatory cytokines (26, 27) via two major proteins with low molecular weights (MWs) of about 14 and 15 KDa (28).

Given the effect of artemisinin on leishmania parasites via cytokine production as well as the immunomodulatory effect of shark cartilage extract, this study is the first to be conducted to assess the efficacy of these biochemicals alone or in combination with glucantime as an antimony drug against *L. infantum *in BALB/c mice. Therefore, the effect of these drugs on Interferon-gamma (IFN-γ) and Interleukin-4 (IL-4) patterns *in L. infantum *infected mice were assessed. In this study, SYBR Green-based RT-qPCR assay were optimized to evaluate parasite loads of visceral leishmaniasis in *L. infantum *infected BALB/c mice and compared using the culturing method.

## Materials and Methods

***Ethics statement***

This project was approved by the Ethical Committee of the School of Medical Sciences of Tarbiat Modares University, adopted from the Helsinki statement (1975) and the Society for Neuroscience, Animal Care, and Use Guidelines (1998), on 27th of April 2015.

***Drug preparation***

Artemisinin (C15H22O5) (MW: 282.4) (Holly Pharmaco, US), was freshly prepared in 1:1 ratio of ethanol and distilled water as a stock solution (16). Mice were given 0.825 mg/kg oral artemisinin daily according to *in vitro *IC50 data results (29). Mice were treated intraperitoneally (IP) with glucantime (Sanofi-Aventis, France: 85 mg/ml, 1 ampule = 5 ml) at 20 mg/kg/day (30).

***Shark cartilage extraction and purification***

Shark cartilage was provided from Bushehr port, southern Iran, by Hassan (31). Briefly, shark cartilage was cleaned, milled, maintained in the freezer overnight and then powdered. The cartilage extract was prepared according to the method described in literature (28). Ten grams of the cartilage powder was extracted in 100 ml of 0.1 M citrate buffer containing 4 M guanidine HCl and a protease inhibitor cocktail (EDTA 6.25 mM, PMSF 1 mM) at pH=5.8 for 48 hr with slight shaking at 2–8^ °^C. The extract was then centrifuged at 100,000 g for 45 min and then dialyzed against PBS (phosphate buffer saline) for 24 hr, and at last, sterilized by Millipore filter equipped with a YM-10 membrane (Sigma-Aldrich Co) (28). The protein concentration of shark cartilage extract was measured by the Bradford protein assay against a standard sample (32). Proteins were separated using SDS polyacrylamide gel electrophoresis. The assay was conducted on 10% polyacrylamide gels according to the protocol. Protein bands were visualized by Coomassie Brilliant Blue (33).

***Promastigotes preparation for infection of BALB/c mice***

*L. infantum* (MCAN/ES/98/LIM-877) was obtained from Kerman University of Medical Science, southern Iran. Promastigotes were cultured in RPMI 1640 (Gibco, US) supplemented with FBS 20% (Fetal Bovine Serum) (Gibco, US) and containing 100 IU/ml of penicillin G plus 100 μg/ml of streptomycin, before being preserved at 18–24 ^°^C till the stationary phase. After 8–10 days of culture, parasites of stationary phase were centrifuged at 2500 rpm for 15 min at 4 ^°^C and washed three times in sterile PBS before being counted and used for animal injection (34).

***Animals, challenge, treatment schedules and follow-up***

A total of 100 female BALB/c mice (mean weight 16–18 g) aged 5–7 weeks, purchased from Pasteur Institute of Iran, were kept at standard temperature (25±5 ^°^C) in a 12-hr day/night cycle and fed standard pellet diet and water, *ad libitum*. The mice were randomly divided into two groups, i.e. control and experimental. Twenty healthy mice were kept as the negative control group (ten mice: uninfected-untreated, ten mice: uninfected-shark cartilage extract fed)**,** whereas 80 mice were peritoneally inoculated with 1×10^7^ stationary phase promastigotes as previously described (34). Treatment schedules and grouping are given in Table 1. Infected mice were kept for three weeks for the establishment of VL. Infection was confirmed in three mice by culturing splenic suspensions and impression smears. A group of 10 infected mice was kept as the positive infected control group and the other 70 mice were divided into 7 even groups and treated with artemisinin, glucantime, and shark cartilage extract either as stand-alone drugs or in combination with each other, as shown in Table 1. The treatment started 21 days after inoculation of promastigotes and was administered consecutively for 28 days. The shark cartilage extract was dissolved in sterile water in a range of concentrations and orally fed to mice at a volume of 0.5 ml/mouse (20 mg/kg) on a daily basis (35, 36). During the treatment period, mice survival was determined in infected groups by postmortem examination. In week 4 post-treatment (day 30), 5 mice of each group were sacrificed for cytokine assay and parasite burden was determined by culturing 30 mg of spleen and liver in RPMI1640 medium, then after one week, the parasites were enumerated according to the modified method of Ahmed *et al* (37). Parasite rates were comparable for both treated and untreated mice groups. Remnant animal groups were followed by 15 weeks of post inoculation.

***Reverse transcription–real-time PCR***

On the basis of properties such as high sensitivity, rapidity, and reproducibility properties, real-time PCR was used to detect *L. infantum* in experimentally infected mouse tissue samples (38).

For RT-PCR analysis, RNA was extracted from 30 mg of spleen and liver tissues, using the RNeasy Mini kit (Qiagen) according to the manufacturer’s instructions. cDNA was prepared with the Quanti Tect Reverse Transcription Kit (Qiagen). Primers for studying the target gene were designed to amplify a 120 bp fragment kDNA Gen of *L. infantum* using the NCBI software. The primer sequences were: Forward 5’-CGCGGGTACCATGCAGGGGACTTGGTTTTC-3’, reverse 5’-CGGGGAATTCTCACTCTTTGCGGATTCTTT-3’. A standard curve was obtained by *L. infantum* promastigotes at stationary-phase, which were gathered, centrifuged, washed twice with PBS, and RNA was extracted from approximately 10^7^ promastigotes. The RNA concentration was measured by spectrophotometric determination of A260.

Real-time RT-PCR was performed using the Light Cycler system with Taq DNA Polymerase 2x Master Mix RED (Viragen Diagnostic), using 2 μl (10 pg) of the cDNA template.

A hot-start method was used to increase specificity. After initial denaturation (10 min at 94 ^°^C), 45 cycles of denaturation for 10 sec at 95 ^°^C, annealing for 10 sec at 54^ °^C, and extension for 25 sec at 72 ^°^C were performed and the PCR was ended by a final elongation at 72^ °^C for 10 min. Each sample was tested in triplicate. The mean cycle threshold (CT) of triplicates in each sample was drawn against the number of parasites (39). 

***Extraction of spleen lymphocytes for cytokine assay***

At the end of treatment (day 30), five mice from each studied group were killed and spleen lymphocytes were extracted for measuring IFN-γ and IL-4 levels. Approximately 2×10^6^ /ml lymphocytes were cultured in 24-well plates in the RPMI1640 cell culture medium (Gibco, USA) containing 10% fetal calf serum (FCS; Gibco, USA), 100 U/ml penicillin and 100 μg/ml streptomycin (Sigma, Germany). The lymphocyte culture was triggered by soluble leishmania antigens (SLA) obtained by re-suspending *L. infantum* promastigotes in sterile PBS at a concentration of about 10^8^/mL. Promastigotes in stationary phase were lysed by six freeze-thaw cycles before being centrifuged at 4 ^°^C for 12 min. The supernatant was gathered and its protein concentration was measured by Bradford assay. SLA at a concentration of 30 μg/ml were added to wells to trigger lymphocytes and the plates were incubated in 5% CO_2_ at 37^ °^C. Supernatants were gathered over 72 hr and stored at -80 ^°^C until use (40). Cytokine levels were measured by enzyme-linked immunosorbent assay kit (Duo Set ELISA, USA & Canada R&D Systems, Inc.) according to the kit procedure.

***Data analysis***

All parasite burden data were shown as the mean ± SD. Differences among groups were analyzed by one-way analysis of variance (ANOVA), and the *Post Hoc Tukey-Krammer* and *Kruskal–Wallis* tests were used for comparison of tests among intragroup. The value of *P<0.05* was considered to be statistically significant. All analyses were conducted using the SPSS version 21 software package for windows. 

## Results

***Extraction and purification of shark cartilage***

**Figure 1:**
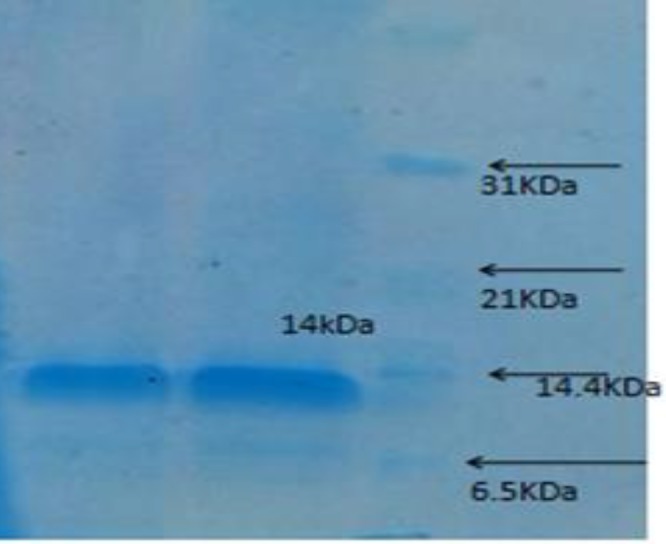
The SDS-PAGE of purified shark cartilage extract

The extracted and partially purified protein of shark cartilage after extraction was dialyzed against PBS on 10% SDS-PAGE showed a 14 kDa band (Figure 1). The purified protein was frozen at -20 ^°^C until used.

***Anti-leishmanial activity of drugs***

**Figure 2:**
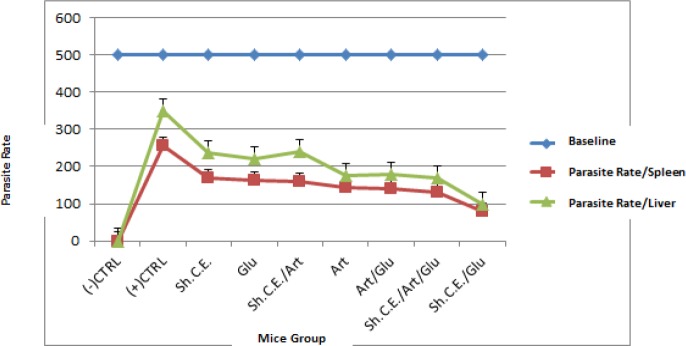
Comparison of parasite rates in spleen (Parasite rate/Spleen) and liver (Parasite rate/Liver) by culturing method. Baseline: The beginning of culture after 21 days of inoculation. At the end of treatment, the parasite counts in the spleen, sere reduced more than in the liver (*P*<0.001)

The *in vivo *anti-leishmanial effects of artemisinin, glucantime, artemisinin plus glucantime, artemisinin plus shark cartilage extract, glucantime plus shark cartilage extract and a combination of artemisinin, glucantime, and shark cartilage extract were shown in Tables 2 and 3. These results showed that the combination of glucantime/shark cartilage extract caused a parasite load reduction more significantly than any of these drugs used as single drug therapy and other groups (*P*<0.001). The herbal drug, artemisinin induced a lower reduction of parasite load when compared to the combination of artemisinin/glucantime (*P*<0.05). There were different values in the anti-leishmanial activity of artemisinin and glucantime (*P<*0.05). However, parasite loads were reduced by all drugs as compared to the control group (*P*<0.001). The highest reduction in parasite numbers was in the combination of shark cartilage extract/ glucantime (Tables 2 and 3). Artemisinin/glucantime and Shark cartilage extract/Artemisinin/Glucantime groups had same means in spleen samples and there were no significant differences between these groups (*P*>0.05). The comparison of parasite burdens in the spleen and liver were shown in Figure 2. The parasite numbers in the spleen, were more reduced at the end of the treatment than in the liver (*P<*0.001).

***The results of real-time PCR***

The forward and reverse primers, which amplify a 120-bp DNA fragment from *L. infantum *kinetoplast minicircles, have already been used. A 10 ng sample of RNA obtained from *in vitro *grown promastigotes of *L. infantum *strain MCAN/ES/98/LIM-877 was used as the template at the start of the PCR assay, and the optimal annealing temperature was determined. Agarose gel electrophoresis of the PCR product proved the amplification of a 120-bp DNA fragment (data not shown). To find non-specific double-stranded reaction products in real-time PCR assay, melting curve analysis was shown at the end of each run. The melting curve of the specific PCR product showed a single peak with the melting temperature of 82 ^°^C, which designates lack of any non-specific products such as primer dimmer.

In order to perform absolute quantification of leishmania parasites and find the limit of detection, the standard curve was prepared using 2-fold serial dilutions of *L. infantum *cDNA. The standard curve was linear over at least six serial dilutions of the parasitic cDNA with the correlation coefficient (R2) value of 0.99 and amplification efficiency of 0.95. The plot is representative of the mean CT values±SD from triplicates against the number of parasites. Inter-assay coefficients of variation of CT values for six 2-fold serial dilutions of *L. infantum *cDNA correspond to 1×10^7^ parasites to 20 parasites in three different runs.

***Evaluation and association between culturing method and real-time PCR***

The number of parasites per 30 mg of the spleen and liver was calculated using real-time PCR by inserting cycle threshold (CT) of samples in a standard curve. Details of CT values and the number of parasites was also counted in culture medium growth (Tables 2 and 3).

***Survival rate***

To evaluate survival rates, the fates of 5 mice in each group were followed up until day 105 (15 weeks) post inoculation. Table 3 summarizes the survival rates of mice of all treatments. No death was recorded in any of the treated groups throughout the experimental period, thus indicating remarkable survival rates in these groups against the positive control group (*P*<0.05). The survival rates of the artemisinin treated group was not different from other treated groups (*P*>0.05), but the rates were significantly higher than those of the positive control group (*P<*0.05). To determine the two distant groups of different average survival rates, multiple comparison tests were carried out using the Dunn-Bonferroni test. The analysis of this test showed that only group 4 (the uninfected mice treated with shark cartilage extract) had significant differences with the positive control group (*P=*0.026).

***IFN-γ and IL-4 pattern***

**Figure 3:**
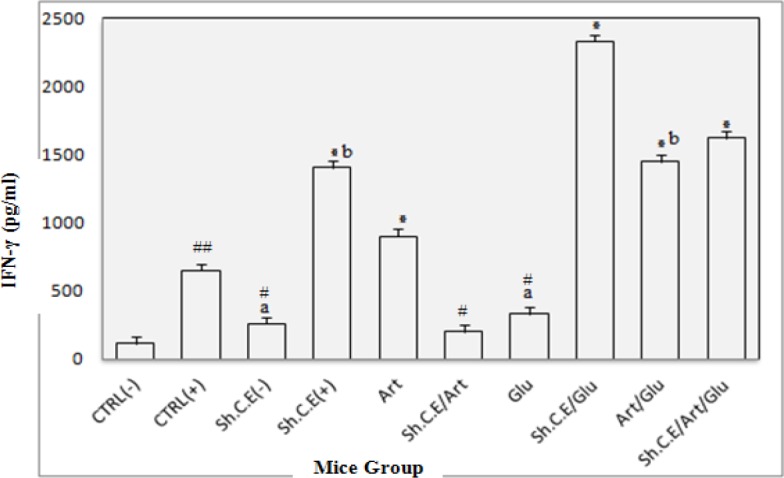
The interferon-gama (IFN-γ) level in the lymphocytes supernatant of the spleen in different groups after 72 hr following stimulation with soluble leishmania antigen (SLA). All of the test groups showed significant differences in spleen compared to the control groups (*P*=0.001). * These groups produced high levels of IFN-γ, significantly compared to the control group (*P*<0.05). # These groups produced low levels of IFN-γ, significantly compared with the positive control group (*P*<0.05). ## (*P*<0.05) versus CTRL(-).^a^ There were no significant differences in IFN-γ levels in these two groups (*P*>0.05). ^b^ There were no significant differences in IFN-γ levels in these two groups (*P*>0.05)

**Figure 4:**
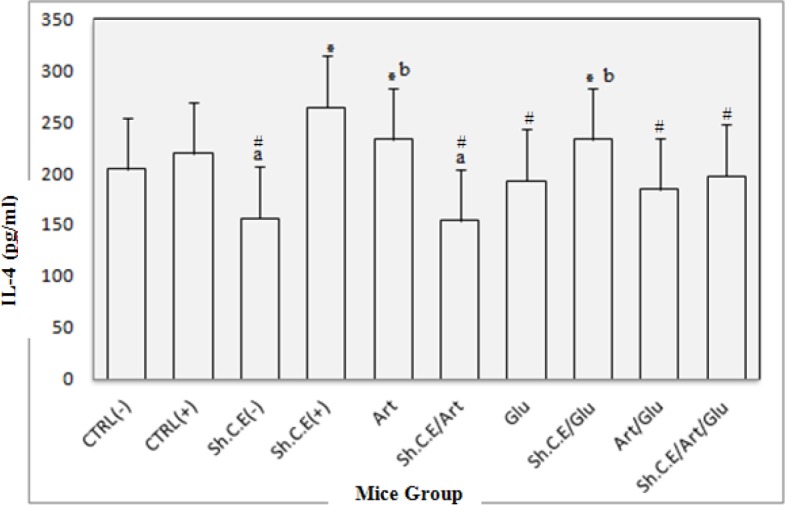
The Interleukine-4 (Il-4) level in the lymphocyte supernatant of the spleen in different groups after 72 hr following stimulation with soluble leishmania antigen (SLA). All groups showed significant differences in IL-4 levels in spleen in test groups compared with the control group (*P*=0.001). * These groups produced high levels of Il-4, significantly compared to the control groups (*P*<0.05). # These groups produced low levels of Il-4, significantly compared to control groups (P<0.05).^a^ There were no significant differences in Il-4 levels in these two groups (*P*>0.05). ^b^ There were no significant differences in IFN-γ levels in these two groups (*P*>0.05)

**Figure 5:**
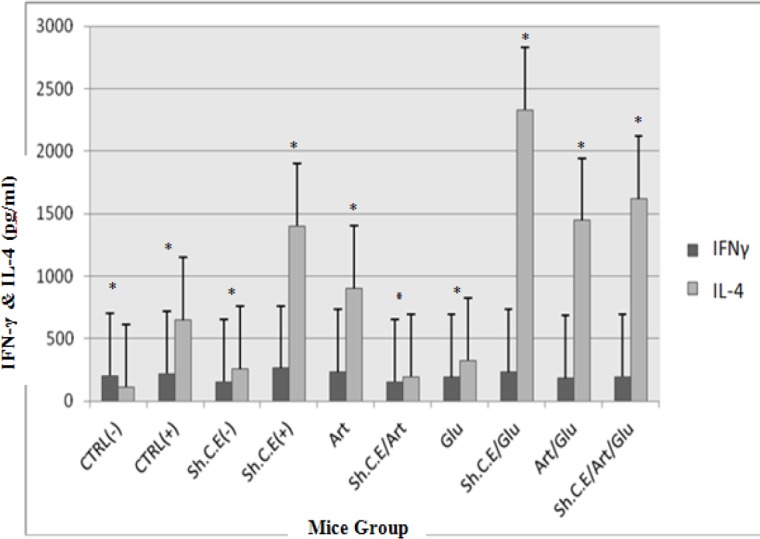
Comparison of Interferon-gama (IFN- γ) and Interleukine-4 (IL-4) in control and test groups in the lymphocyte supernatant of the spleen in different groups after 72 hr following stimulation with soluble leishmania antigen (SLA). *There were significant differences of IFN- γ and IL-4 in the same groups (*P*<0.05)

Assessment of Interferon-Gama and Interleukin-4 cytokine levels in the test and control groups was carried out by ELISA reader system 72 hr after culture. Results were compared with INF-γ and IL-4 standard curves and control groups. The results presented in Figure 3 and Figure 4 show a significant increase of IFN-γ and IL-4 compared to the control group (*P<*0.05) after treatment. However, IL-4 increased more slowly than IFN-γ. Artemisinin makes the INF-γ and IL-4 cytokine levels increase more sharply in treating mice rather than in control groups. The positive control group as well as uninfected mice treated with shark cartilage extract produced low levels of IFN-γ, but infected mice treated with shark cartilage extract or its combination with glucantime showed significantly higher levels of IFN-γ as in Figure 3 (*P<*0.05). There was the remarkable difference between IFN-γ levels induced by a combination of artemisinin and shark cartilage extract treated group with those in the control group. Unexpectedly, the lowest IFN-γ levels were in shark cartilage extract and artemisinin combination. Interleukin-4 levels were lower than IFN-γ levels between the groups (Figure 5). The results showed that IL-4 levels induced by shark cartilage extract were significantly different in the test group compared with the control group. However, IL-4 levels showed a significant difference with the negative control (*P<*0.05). There was a remarkable difference between IFN-γ levels induced by a combination of artemisinin and shark cartilage extract treated group with those in the control group. Unexpectedly, the lowest IFN-γ levels were in shark cartilage extract and artemisinin combination. IL-4 levels were lower than IFN-γ levels between the groups (Figure 5). The results showed that IL-4 levels induced by shark cartilage extract were significantly different in the test group compared with the control group. However, IL-4 levels showed a significant difference from the negative control group when compared with the positive control group (*P=*0.001).

## Discussion

A depressed immune system with disseminated leishmaniasis is the most important problem in leishmaniasis control in endemic areas (41). In this situation, a positive contribution of effective host defense mechanisms to help chemotherapeutic drugs to boost the depressed immune systems is deemed to be necessary. Herbal drugs and their combination with currently available drugs may not only reduce cost, toxicity, and duration of treatments but also open a promising window to achieve effective leishmaniasis control (42). Studies have already shown that artemisinin has not only therapeutic effects against experimental models of leishmaniasis but also immunomodulatory effect (19). Also, previous studies on shark cartilage have shown immunological enhancement of CD4/CD8 in murine tumor cells (31). Another *in vitro *study showed that stimulation with shark cartilage triggers the production of Th1-type inflammatory cytokines such as IL-1β, IL-2, TNF-α, and IFN-γ (27).

This is the first experimental *in-vivo *study of the synergistic effect of combination therapy between artemisinin, glucantime and shark cartilage extract. In combination therapy, mechanisms of action, pharmacodynamics and pharmacokinetics of anti-leishmanial drugs must be noticed. Anti-leishmanial drugs have different activities in pharmacokinetics, so it is expected to have different behaviors (43).

In this study, the most sensitive gene, the kDNA gene, was used for evaluating the effect of target drugs on visceral leishmaniasis in BALB/c mice by real-time PCR.

Similar to other drugs, *in vivo *interactions between artemisinin and other studied drugs can occur during absorption, tissue distribution, or elimination of misuse drug. However, leishmania is an intracellular parasite in the organs of the reticuloendothelial system, so the volume of tissue distribution and uptake into macrophages are important. Furthermore, time of usage, route of administration, dosage and adverse effects of drugs are effective in pharmacokinetics synergism in drugs. According to the pharmacokinetics of artemisinin and shark cartilage, these drugs were given orally, but glucantime was injected parenterally in this study. Artemisinin has a low solubility in water or oil and is usually administered orally (44).

In this study, the activity of artemisinin was also enhanced when it was combined with the standard dose of glucantime, but artemisinin-shark cartilage extract combination showed no potentiation synergism. Conversely, the combination of glucantime and shark cartilage extract had the most positive effect. Surprisingly the combination of the three, artemisinin-glucantime-shark cartilage extract, showed noticeable synergism. Other studies have shown the synergistic effect of artemisinin with antibiotics in mice against lethal live *Escherichia coli *challenge. The endoperoxide bridge is necessary for the artemisinin activity, the first activated by intraparasitic heme iron (ferrous form), which break up the endoperoxide ring, could transfer an oxygen atom from the peroxide group to a chelated iron ion and the last produces oxygen species; these free radical products intervene to kill the malaria parasite by alkylation and destruction of parasite proteins (45). Leishmania parasites are ironophillic parasites and also scavenge iron from its host macrophage so administration of artemisinin to leishmania-infected BALB/c mice eliminates intracellular amastigotes via production of the iron–artemisinin component (17).

Recent research suggests that basic proteins in shark cartilage are absorbed via the intestinal tract. The action mechanisms of shark cartilage have been proven in anti-cancer mechanisms to be through direct toxicity against tumor cells by inhibition of tumor angiogenesis in animal models with glycoproteins and stimulation of the immune system. Ongoing research has reported that shark cartilage acts as a scavenger of reactive oxygen species, interferes with cellular adhesion, and inhibits calcium channels (22).

**Table 1:** The studied mice groups in this study (10 mice in each group)

	**Mice groups (treated with)**	**Abbreviation **
Uninfected mice	Uninfected-untreated	CTRL (-)
	Shark cartilage extract	Sh.C.E. /uninfected
Infected mice	Shark cartilage extract	Sh.C.E./infected
	Artemisinin	Art
	Glucantime	Glu
	Artemisinin- glucantime combination	Art/Glu
	Artemisinin- shark cartilage extract combination	Sh.C.E./Art
	Glucantime-shark cartilage extract combination	Sh.C.E./Glu
	Artemisinin- glucantime-shark cartilage extract combination	Sh.C.E./Art/Glu
	Infected-untreated	CTRL(+)

**Table 2:** Detection of the parasite count of spleen sample in *Leishmania* *Infantum* infected Balb/c mice at 7 weeks post-infection using Real-Time PCR and culturing

Mice group	Quantitative real-time PCR		٭٭Mean count of parasites/30 mg of spleen tissue (culturing)(×10^6^)
	٭Count of parasites/30 mg of spleen tissue	Mean ± SD of CT	
Sh.C.E./infected	28500	27.51± 0.17	1.88± 2.3
Art	21000	25.47± 0.33	1.43± 2.2
Glu	25000	23.92± 0.69	1.63± 3.3
Art/Glu	21500	24.89± 0.15	1.39± 2.9
Sh.C.E./Art	27000	20.89± 0.67	1.79± 2.9
Sh.C.E./Glu	5100	32.23± 0.37	0.78± 2.3
Sh.C.E./Art/Glu	18500	25.90± 0.28	1.32± 1.1
Control (Infected-untreated)	35500	21.73± 0.21	2.57± 2.7

٭ *P<0.05 *There are significant values between test and control groups

٭٭ *P<* *0.01 *There are significant values between test and control groups

**Table 3:** Detection of the parasite count of liver sample in *Leishmania infantum *infected Balb/c mice at 7 weeks post-infection using real-time PCR and culturing

Mice group	Quantitative real-time PCR		٭٭ Mean count of parasites/30 mg of liver tissue(culturing) (×10^6^)
	٭Mean count of parasites /30 mg of liver tissue	Mean ± SD of CT(Cycle treshold)	
Sh.C.E./infected	31000	22.92± 0.34	2.36± 2.6
Art	25500	25.71± 0.39	1.77± 1.1
Glu	29500	23.17± 0.67	2.21± 3.5
Art/Glu	19500	30.91± 0.75	1.79± 1.1
Sh.C.E./Art	29500	27.50± 0.21	2.39± 1.9
Sh.C.E./Glu	7500	35.17± 0.18	0.97± 2.8
Sh.C.E./Art/Glu	17500	32.18± 0.26	1.69± 1
CTRL(Infected-untreated)	36500	22.10± 0.91	3.50± 3.2

٭ *P<* *0.05 *There are significant values between test groups and control groups

٭٭ *P<* *0.01* There are significant values between test groups and control groups

Sh.C.E: shark cartilage extract; Art: artemisinin; Glu: glucantim; Art/Glu: artemisinin- glucantim combination; Sh.C.E./Art: shark cartilage extract- artemisinin combination; Sh.C.E./Glu: shark cartilage extract- glucantim combination; Sh.C.E./Art/Glu: shark cartilage extract- artemisinin- glucantim combination

Sh.C.E: shark cartilage extract; Art: artemisinin; Glu: glucantim; Art/Glu: artemisinin- glucantim combination; Sh.C.E./Art: shark cartilage extract- artemisinin combination; Sh.C.E./Glu: shark cartilage extract- glucantim combination; Sh.C.E./Art/Glu: shark cartilage extract-artemisinin- glucantim combination

Sh.C.E: shark cartilage extract; Art: artemisinin; Glu: glucantim; Art/Glu: artemisinin- glucantim combination; Sh.C.E./Art: shark cartilage extract- artemisinin combination; Sh.C.E./Glu: shark cartilage extract- glucantim combination; Sh.C.E./Art/Glu: shark cartilage extract- artemisinin- glucantim combination

Sh.C.E: shark cartilage extract; Art: artemisinin; Glu: glucantim; Art/Glu: artemisinin- glucantim combination; Sh.C.E./Art: shark cartilage extract- artemisinin combination; Sh.C.E./Glu: shark cartilage extract- glucantim combination; Sh.C.E./Art/Glu: shark cartilage extract- artemisinin- glucantim combination

In our study, glucantime showed higher efficacy when co-combined with artemisinin and especially when co-combined with shark cartilage extract against murine visceral leishmaniasis. This finding emphasizes that the effect of combination drug therapy is more than each its components used alone in inhibiting parasite growth in BALB/c mice. In this study, artemisinin showed higher efficiency compared to glucantime. Treatment with artemisinin led to a significant reduction of parasite burdens in mouse liver in comparison to glucantime alone, though the result was not the same in the spleen.

The negative result of artemisinin-shark cartilage extract co-combination may be related to the contrast effect of artemisinin and shark cartilage on reactive oxygen species or calcium channel inhibition. On the other hand, it may be related to the low bio half-life of artemisinin (2–5 hr), and it needed to be used several times daily. In this study, we used artemisinin two times a day, but other studies showed that when used 4 times per day for treating visceral leishmaniasis, it could have resulted in better therapeutic effects (17).

The mechanical action of pentavalent antimonial is not clear, but some studies showed DNA damage after treatment with meglumine antimoniate (20 mg/kg for 20 days) in BALB/c mice infected by *L*. *infantum*. Also, the oxidative stress enzymes were activated in the infected animals. On the other hand, the treatment of *L. infantum *infection with meglumine antimoniate induces oxidative stress-derived DNA damage (46).

The authors have already demonstrated the parasiticidal activity of artemisinin both *in vitro *and *in vivo *using experimental models of cutaneous leishmaniasis. These studies showed a significant decrease in parasitic burdens in the test cultures. Also, Sen *et al.* proved the anti-leishmanial activity of artemisinin in murine experimental visceral leishmaniasis. They reported splenic weight and parasite burden reduction in BALB/c mice equal to 82.6% and 86.0%, respectively (19). We also studied the efficacy of artemisinin alone or combined with glucantime (Art/Glu) or artemisinin with glucantim and shark cartilage extract (Art/Glu/ShCE) in experimental models of VL. The current study showed the effect of artemisinin and shark cartilage extract as an immunomodulator.

The follow up of mice survival during the course of this study showed high survival rate of the mice treated with either artemisinin-glucantime or artemisinin-glucantime-shark cartilage extract, which may depend on their effective reduction of parasite burdens. This is another indication of the therapeutic value of the drugs in controlling leishmania parasite growth. Also, we did not notice any side effects following administration of the drugs at given dosages before the end of treatment. Ghaffarifar *et al.* showed that higher survival rates of mice infected with *Leishmania major *were related to those treated with artemisinin ointment (16).

Two cell populations determine leishmanial condition: Th1 (IFN-γ, IL-2) in the protective condition and Th2 (IL-4, IL-10, and IL-13) in progressive infection. In experimental VL, IFN-γ and IL-12 cause the protective condition while IL-4 and IL-10 enhance the progression of infection (47).

IFN-γ is an important glycoproteineic cytokine that is essential for an immune cellular response against leishmania, which activates macrophage cells in antigen presentation to T cells, nitric oxide production and Th1 differentiation (48). In this study, drugs triggered production of both IFN- γ and IL-4, but IFN-γ was produced more than Il-4. We showed that treatment with artemisinin enhances the ability of lymphocytes of infected mice to produce INF-γ. We also showed a significant IFN-γ response in the spleen cell cultures of test groups compared with those in the control group. Shark cartilage extract alone induces production of both IFN-γ and IL-4 in treating mice at levels differing from the control group. The findings of this study correspond to others studies, demonstrating that shark cartilage extract influences *in vitro *production of IL-4 and IFN-γ by spleen or lymph node cells (28). A very high increase in the IFN- γ level in shark cartilage extract and glucantime combination and very low IFN- γ level in shark cartilage extract with artemisinin combination were the noticeable point in this study. Immunomodulation of anti-leishmanial drugs such as glucantime, amphotericin B, and miltefosine was proven by some studies.

Our study had no limitation because it necessarily distinguishes between live and dead parasites. The levels of leishmania RNA indicated the parasite loads of live parasites. In summary, we have for the present study used a very sensitive method for detection of leishmania, and it can be applied to investigate several other different species of the parasite.

## Conclusion

Shark cartilage extract could increase glucantime activity, but decrease artemisinin activity. Also, immunomodulation can increase anti-leishmanial effects of antimonial drugs. This study data showed, the combination of glucantime and shark cartilage extract had the most positive effect rather than glucantime and artemisinin or artemisinin-glucantime-shark cartilage extract combination. These combinations were able to be used in antimony-resistant VL or antimony-failure treatment cases in Iran. However, further studies to show the basis of interactions of these drugs, including water-soluble formulations of artemisinin will be conducted. This study also proved that artemisinin alone as an immunomodulator can be a candidate for further evaluation as a chemotherapeutic agent for the treatment of leishmaniasis.
